# Supplementary material for: Mpp23Aa/Xpp37Aa Insecticidal Proteins from Bacillus thuringiensis (Bacillales: Bacillaceae) Are Highly Toxic to Anthonomus grandis (Coleoptera: Curculionidae) Larvae
Source: Toxins (Basel). 2023 Jan 8;15(1):55. doi: 10.3390/toxins15010055 (PMC9865532; doi:10.3390/toxins15010055)
Supplement: Supplementary file 1 [file toxins-15-00055-s001.zip › toxins-2129256-supplementary.pdf]

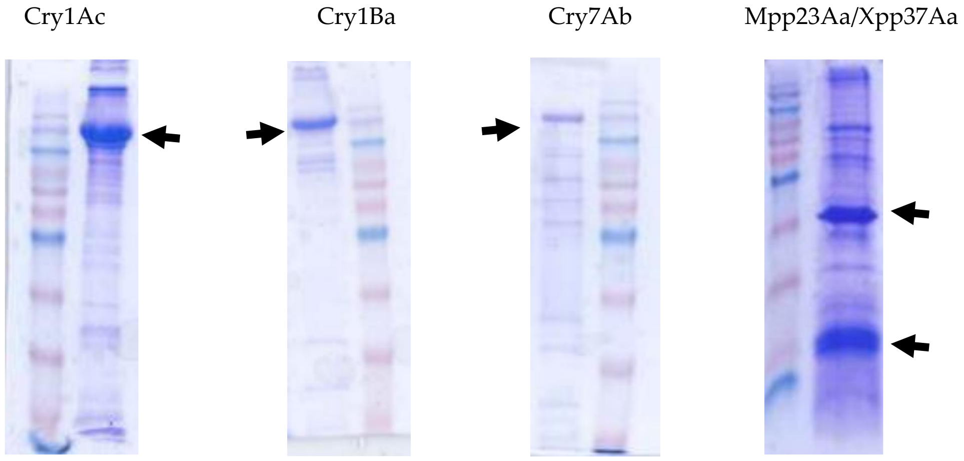

Figure S1. SDS–polyacrylamide gel electrophoresis of insecticidal proteins used in the toxicity tests: Cry1Ac, Cry1Ba, Cry7Ab, and Mpp23Aa/Xpp37Aa. BlueStar prestained protein marker (bands in kDa: 180, 130, 100, 75, 63, 48, 35, 28, 17, 10). Bands were stained with Coomassie blue. The Bt insecticidal protein bands are indicated by an arrow.
